# The clinical effects of probiotic supplementations on metabolic syndrome: a systematic review and meta-analysis

**DOI:** 10.3389/fnut.2026.1888320

**Published:** 2026-09-14

**Authors:** Min Chen, Jing Li, Shumin Li

**Affiliations:** Department of Internal Medicine, Ningbo No.6 Hospital, Zhejiang, China

**Keywords:** glucose, HDL, LDL, metabolic syndrome, probiotics, total cholesterol, triacylglycerol

## Abstract

**Background/Objectives:**

Probiotics have attracted much attention due to their ability to modulate nutrient metabolism or immunomodulatory. This study aims to evaluate the efficacy of probiotic supplementation in the treatment of metabolic syndrome.

**Methods:**

PubMed, Web of Science, Embase, and Cochrane Library were systematically searched searched for eligible clinical trials published from database inception to July 2024. STATA/SE 14.0 software was used for statistical analysis.

**Results:**

A total of 12 clinical trials with 633 patients diagnosed with metabolic syndrome were included. The intervention group received probiotic supplementations. The control group received placebo. HDL level was significantly higher with probiotic supplementation than that in the placebo group (WMD = 1.63 mg/dL, 95%CI: [−0.10, 3.36], *p* = 0.065; I^2^ = 89.7%, *p* < 0.001). LDL level was significantly lower with probiotic supplementation than that in the placebo group (WMD = −0.59 mg/dL, 95%CI: [−1.17, −0.02], *p* = 0.044; I^2^ = 33.4%, *p* = 0.123). For age ≤50 years, probiotics supplement inhibited the serum triglyceride levels (WMD = −10.75 mg/dL, 95%CI: [−22.17, 0.67], *p* = 0.065; I^2^ = 77.8%, *p* < 0.001). Significant difference was observed in glucose (WMD = −0.58 mg/dL, 95%CI: [−1.15, −0.02], *p* = 0.043; I^2^ = 0.0%, *p* = 0.966), whereas no significant difference was observed in total cholesterol between two groups (WMD = 1.80 mg/dL, 95%CI: [−5.37, 8.98], *p* = 0.622; I^2^ = 87.4%, *p* < 0.001).

**Conclusion:**

This study indicates that probiotic supplementation shows potential therapeutic benefit in metabolic syndrome.

**Systematic review registration:**

https://www.crd.york.ac.uk/prospero/, identifier CRD42025628477.

## Introduction

Metabolic syndrome (MetS), also known as syndrome X or insulin resistance syndrome (IRS), is the failure of systemic metabolism functions that are related to elevated blood pressure, elevated triglycerides and lowered high-density lipoprotein cholesterol, elevated fasting glucose, and central obesity based on International Diabetes Federation and the American Heart Association/National Heart, Lung, and Blood Institute (1). MetS is a term that serves as an umbrella of risk factors for individuals to be at an increased risk of disease rather than a disease, which increases cardiovascular morbidity and mortality as well as overall mortality (2). MetS also contributes to the prevalence of type 2 diabetes, coronary diseases, stroke, and other disabilities, contributing to cost trillions including the cost of health care and loss of potential economic activity (3). The epidemic of MetS often parallels the incidences of obesity and type 2 diabetes (3). Center of Disease Control and Prevention (CDC) reported that the United States reported a 35% increase in MetS prevalence from 1980s to 2012 (4). Due to unawareness, MetS actual prevalence is three times higher, accounting for one-third of the American adult population (3). Unfortunately, this trend is also widespread in China (5). In view of aging population, MetS has become a public health problem worthy of attention.

MetS has a complex pathology. Previous studies have proposed several etiological factors, such as poor lifestyles, genetic susceptibility, environmental pollutants exposure (6). Among these factors the gut microbiome has recently taken center stage, interconnecting the above factors and playing a central role in modulating human health. The trillions of microbial cells, as “second genome” are responsible for over 98% of the genetic responses and maintain homeostasis and immune function (7). Beyond the GI tract, microbiome mediates a variety of critical communications between the gut, enteric nervous system (ENS), and the brain, regulating the systemic metabolism process (8). Hence, change in the microbial ecosystem could possibly contribute to the development of metabolic diseases via microbiota-induced metabolites, amongst others short-chain fatty acids (SCFA), hormones, neurotransmitters, trimethylamine N-oxide and secondary bile acids (9–11). The gut microbiota can be modulated by probiotic supplementation. and it has been documented in of many therapeutic trials of many diseases that when administered in adequate doses confer a health benefit to the host (12). Nowadays, probiotics have been successfully used in the prevention and treatment of obesity, diabetes mellitus, irritable bowel syndrome, even multiple sclerosis and Alzheimer’s disease (13), by inhibition of undesirable programming processes, which is referred to as reprogramming (14). However, the clinical evidence on the efficacy of probiotic supplementation for MetS remains controversial and inconsistent: some studies have confirmed that probiotics can improve dyslipidemia and insulin resistance in MetS patients (15, 16), while others have found no significant therapeutic effect (17, 18). The main reasons for this inconsistency include the heterogeneity of probiotic interventions, differences in study population characteristics and inconsistent intervention durations (16). In particular, age, as an important demographic factor, is closely related to the changes in gut microbiome structure and metabolic function of the body. However, the optimal age threshold for predicting the response to probiotic supplementation in MetS remains unknown. Therefore, we performed an exploratory subgroup analysis based on age categories to investigate whether age-related differences may contribute to heterogeneity among studies. Based on the above research deficiencies, this study comprehensively evaluated the effects of probiotics on core metabolic indicators (glucose, HDL-C, LDL-C, TC, TG) of Mets, and performed subgroup analysis based on age (<50 years and >50 years) to explore the age-specific efficacy of probiotics. At the same time, this study further analyzed the sources of heterogeneity in the included studies, and put forward targeted solutions for the high heterogeneity of probiotic intervention schemes, aiming to provide more precise evidence-based medical evidence for the clinical application of probiotics in the treatment of MetS, and clarify the research direction for subsequent clinical trials.

## Materials and methods

### Literature search

This meta-analysis was conducted based on the Preferred Reporting Items for Systematic Reviews and Meta-Analyses (PRISMA 2020) guidelines (19), followed the PICOS principle to formulate the research question and screening criteria: Participants (P): Patients diagnosed with Met according to IDF, AHA/NHLBI or other internationally recognized diagnostic criteria, regardless of gender, race and disease duration; Intervention (I): The intervention group received any form of probiotic supplementation (e.g., probiotic powder, fermented milk, yoghurt, cheese) alone or as a synbiotic (probiotics + prebiotics); Control (C): The control group received a placebo, conventional food without probiotics or no intervention: Outcomes (O): Primary outcome indicators included core metabolic indicators of MetS: fasting blood glucose, HDL-C, LDL-C, TC, TG: Study design (S): Randomized controlled trials (RCTs) and comparative retrospective cohort studies evaluating probiotic supplementation in patients with metabolic syndrome. The study protocol was registered in PROSPERO (registration number: CRD42025628477).

### Search strategy

PubMed, EMBASE, the Cochrane library, and Web of Science database were comprehensively searched for relevant studies from their inception until July 2024, The search strategy (see Supplementary Table 1) was constructed by combining Medical Subject Headings (MeSH/exp) and free-text terms (title/abstract/keyword), with core terms including probiotics, probiotic, probiotic agent, Metabolic Syndrome and their synonymous expressions. All databases adopted the combined retrieval of probiotic-related and Metabolic Syndrome-related terms, with human study population filters applied as appropriate. The search and study selection process was performed independently by two investigators (Min Chen & Shumin Li). It included the analysis of titles/abstracts followed by the full texts. Discrepancies and disagreements in the results were examined by a third investigator (Jing Li).

### Inclusion and exclusion criteria

Two investigators (Min Chen & Shumin Li) independently screened the literature according to the pre-set inclusion and exclusion criteria, and the screening process was divided into two stages: title/abstract screening and full-text screening. Discrepancies in the screening results were resolved through discussion with a third investigator (Jing Li). The inclusion criteria were (1) included patients with metabolic syndrome, (2) The intervention measure was probiotic supplementation (single strain or composite strain) with clear strain, dose and intervention duration; (3) the control group without probiotics, (4) the outcomes contained metabolites, such as glucose, high density lipoprotein (HDL), low density lipoprotein (LDL), total cholesterol, and triacylglycerol, (5) full texts comparative clinical studies (RCTs or retrospective cohort studies).

The exclusion criteria were (1) Non-RCT studies (e.g., conference abstracts, case reports, cohort studies, animal experiments, review articles, study protocols); (2) Repeated published studies or duplicate data, (3) unretrievable texts/missing key data, or (4) combined with other active treatments.that may affect the outcome indicators;

### Data extraction and quality assessment

Two investigators (Min Chen & Shumin L) independently extracted data from the eligible studies using a pre-designed data extraction form, and cross-checked the extraction results. The extracted content included: (1) Basic study information: author’s name, publication year, country, study design, registration number; (2) Study population characteristics: sample size (intervention group/control group), age, gender ratio, baseline Mets severity; (3) Intervention details: probiotic strain, viable cell concentration, intervention form, intervention duration, follow-up time; (4) Outcome indicators: mean and standard deviation (SD) of fasting blood glucose, HDL-C, LDL-C, TC, TG in the intervention group and the control group before and after intervention; (5) Risk of bias assessment indicators: randomization method, blinding method, incomplete outcome data, selective reporting, other biases. The methodological quality of randomized controlled trials was assessed using the Cochrane Risk of Bias 2.0 tool, whereas the retrospective comparative study was evaluated using the ROBINS-I tool, in accordance with Cochrane recommendations for non-randomized studies of interventions. ROB 2.0 covers five domains of bias: bias arising from the randomization process, bias due to deviations from intended intervention, bias due to missing outcome data, bias in measurement of the outcome, and bias in selection of the reported outcome. ROBINS-I evaluates seven domains of bias: bias due to confounding, bias in the selection of participants, bias in the classification of interventions, bias due to deviations from intended interventions, bias due to missing data, bias in measurement of outcomes, and bias in selection of the reported result. For both tools, each domain and the overall quality were classified as high, low, or some concerns. Two investigators independently assessed the risk of bias of the included studies using the appropriate assessment tool according to study design. Disagreements between them were resolved by consulting with a third investigator.

### Statistical analysis

The primary outcomes of this meta-analysis were fasting glucose, HDL-C, LDL-C, total cholesterol, and triglyceride levels. Overall pooled analyses were performed for each outcome whenever sufficient data were available. Age subgroup analysis (<50 vs. >50 years) was prespecified based on the biological hypothesis that age-related alterations in gut microbiota composition and metabolic function may modify the response to probiotic supplementation (20). Additional subgroup analyses according to BMI, intervention duration, and control group type, together with meta-regression analyses, were performed as exploratory analyses to investigate potential sources of heterogeneity. The meta-analysis was performed using the STATA/SE 14.0 software (StataCorp, College Station, Texas, USA). Weighted mean difference (WMD) and 95% confidence intervals (CIs) were used to compare the outcomes. The study used χ^2^ and I-squared (I^2^) to evaluate the heterogeneity. The random-effect model was adopted if the *P* ≤ 0.05 and I^2^ ≥ 50%, which meant existing heterogeneity among studies model. Otherwise, the fixed-effect model was applied (21). Publication bias was assessed using funnel plots and the Begg rank correlation (22). If significant bias was present, trim-and-fill analysis was used to judge whether the publication bias had an impact on the outcomes. Sensitivity analysis by leave-one-out method was used to test the robustness of the results. *P* < 0.05 indicated statistical significance.

## Results

### Identification and selection of the eligible studies

A total of 4,308 studies were identified through searches of the four electronic databases. After removing 1,124 duplicate records, 3,184 studies were screened based on title and abstract, and 3,165 studies were excluded (including 602 reviews, 137 meta-analyses, 171 study protocols, 137 meeting, 1,750 irrelevant studies and 368 for other reason). A total of 19 studies were further assessed for eligibility by full-text review, and 6 studies were excluded (3 studies with incomplete key outcome data, 2 studies with non-probiotic control group, 1 study with combined intervention of hypolipidemic drugs). Finally, 12 RCTs and one retrospective trial involving 673 patients with MetS were included in this meta-analysis. The detailed literature screening process and results are shown in Figure 1

**FIGURE 1:**
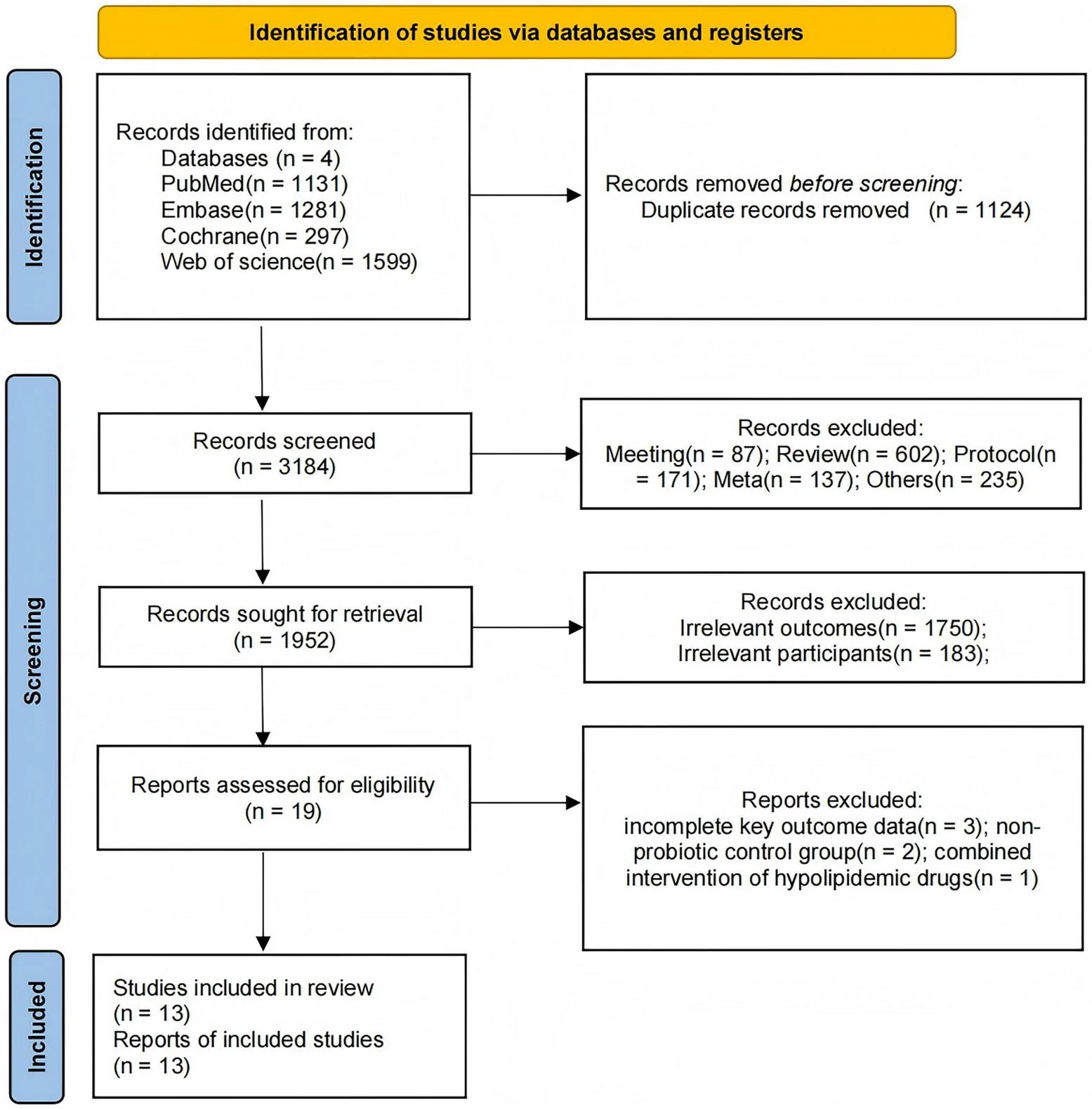
Study selection process.

### Study characteristics and quality assessment

The 12 studies that met the inclusion criteria were published between 2012 and 2022. Four studies were conducted in Brazil, four in Iran, and one each in Italy, Austria, Canada, and Russia. The majority of the study population were middle-aged. The participants’ demographic characteristics in the included studies are summarized in Table 1. The methodological quality of the included studies was assessed using ROB 2.0 (Supplementary Figures 1, 2) or NOS (Supplementary Figures 3, 4). Overall, most studies were judged to have a low risk of bias.

**TABLE 1:** Characteristics of the included studies.

References	Time	Country	Intervention (n)	Control (n)	Age (years)	Outcomes	Types of probiotics
(17, 18, 23, 27, 31–39)	2012.01–2012.10	Brazil	FM (12)	NFM (12)	Control: 63 (60.5–75.7) Intervention: 62 (58.3–67)	Anthropometric measurements, Blood pressure, and Laboratory parameters	Lactobacillus plantarum Lp 115
(23, 31)	–	Italy	Synbiotic treatment (30)	Placebo (30)	Control: 71 ± 3 Intervention: 72 ± 3	Anthropometric measurements, blood pressure, Laboratory parameters, Quality of life, Endothelial reactivity and Safety	Lactobacillus plantarum PBS067, L. acidophilus PBS066, L. reuteri PBS072
(23,32)	2013.03–2013.05	Brazil	Probiotic milk (26)	Untreated (25)	–	Anthropometric measurements, blood pressure, and laboratory parameters	Bifidobacterium animalis ssp. lactis HN019
(32, 33)	2019.04–2019.10	Iran	Synbiotic supplement (30)	Placebo (30)	Control: 40.6 ± 1.13 Intervention: 42.33 ± 1.49	Anthropometric measurements, blood pressure, and laboratory parameters	Lactobacillus casei, L. acidophilus, L. rhamnosus, L. bulgaricus, Bifidobacterium breve, B. longum, Streptococcus thermophilus
(33, 34)	–	Iran	Synbiotic supplementation (19)	Placebo (19)	Control: 46.05 ± 10.1 Intervention: 47.52 ± 9.1	Anthropometric measurements, blood pressure, dietary intake and laboratory parameters	Lactobacillus casei, L. acidophilus, L. rhamnosus, L. bulgaricus, Bifidobacterium breve, B. longum, Streptococcus thermophilus
(34, 35)	2016.11–2017.03	Iran	Synbiotic supplementation (52)	Placebo (56)	Control: 45.64 ± 9.33 Intervention: 42.77 ± 8.35	Anthropometric measurements, blood pressure, dietary intake, physical activity and laboratory parameters	Lactobacillus casei, L. acidophilus, L. rhamnosus, L. bulgaricus, Bifidobacterium breve, B. longum, Streptococcus thermophilus
(35, 36)	2016.10–2017.03	Iran	Probiotic yoghurt (22)	Regular yoghurt (22)	Control: 44.55 ± 5.70 Intervention: 44.05 ± 6.60	Anthropometric measurements, blood pressure, dietary intake and laboratory parameters	Lactobacillus acidophilus La5 + Bifidobacterium lactis Bb12
(36)	2014.08–2015.06	Brazil	Synbiotic supplementation (23)	Placebo (22)	Control: 49.5 (39.5–59.5) Intervention: 47.0 (41.0–53.0)	Anthropometric measurements, heart rate, and laboratory parameters	Lactobacillus acidophilus La-5
(18, 37)	–	Brazil	Probiotic milk (24)	Curd drinks (24)	Control: 42 ± 14 Intervention: 44 ± 10	Anthropometric measurements, blood pressure and laboratory parameters	Kefir(Composite lactic acid bacteria, acetic acid bacteria, yeast)
(18, 38)	–	Austria	Probiotic supplementation (13)	Placebo (15)	Control: 54.5 ± 8.9 Intervention: 51.5 ± 11.4	Gut permeability, endotoxin determination, oxidative burst, anthropometric measurements, laboratory parameters	Lactobacillus casei Shirota
(38)	2017.01–2017.03	Canada	FSY (44)	LFY (43)	Control: 45.6 ± 8.7 Intervention: 45.4 ± 8.9	Anthropometric measurements, blood pressure, and laboratory parameters	Bifidobacterium animalis ssp. lactis Bb-12
(27, 39)	2011.01–2011.03	Russian	Probiotic cheese (25)	Control cheese (15)	/	Anthropometric measurements, blood pressure, and laboratory parameters	Lactobacillus plantarum TENSIA (DSM 21380)
(40)	NR (trial registered before 2019; intervention 11 weeks)	China	Probiotic combination (21)	Placebo (19)	Control: 51.17 ± 8.43 Intervention: 49.04 ± 14.64	Anthropometric measurements, FBG, fasting insulin, HbA1c, TG, TC, HDL-C, LDL-C, gut microbiota, SCFAs	Bifidobacterium adolescentis CCFM8630 + Lactobacillus reuteri CCFM8631 (1 × 10^10^ CFU/day)

FM, fermented milk; NFM, non-fermented milk; FSY, fortified yogurt; LFY, low-fat conventional yogurt.

### Laboratory parameters

#### Glucose

Nine RCTs and one retrospective trial reported the effect of probiotic supplementation on the glucose level. The results indicate that the glucose level was comparable between probiotic supplementation and placebo (WMD = −0.58 mg/dL, 95%CI:[−1.15, −0.02], *p* = 0.043; I^2^ = 0.0%, *p* = 0.966) (Figure 2), showing single probiotic supplementation may have no significant effect on glucose level.

**FIGURE 2:**
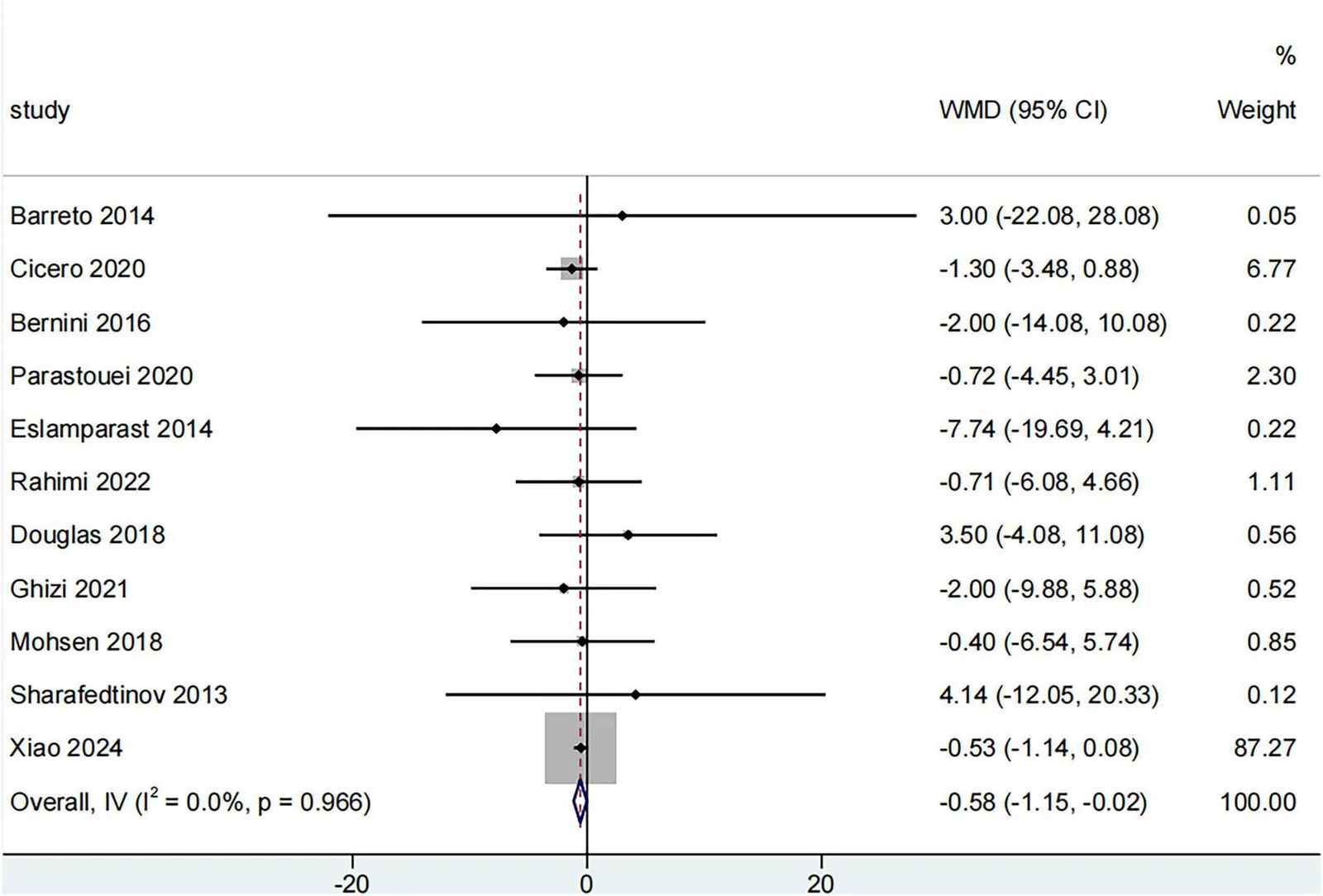
Forest plot of the glucose (overall).

#### HDL

Ten RCTs and one retrospective trial reported the effect of probiotic supplementation on the HDL level. The results indicate that the HDL level was higher with probiotic supplementation than that in the placebo group (WMD = 1.63 mg/dL, 95%CI: [−0.10, 3.36], *p* = 0.065; I^2^ = 89.7%, *p* < 0.001) (Figure 3). Then, a subgroup analysis of the effect of probiotic supplementation on the HDL level was performed according to age. Three studies reported the age >50 years, and eight studies reported age ≤50 years. Probiotic supplementation significantly increased HDL level in patients with age ≤50 years (WMD = 2.08 mg/dL, 95%CI: [0.08, 4.07], *p* = 0.042; I^2^ = 91.2%, *p* < 0.001) rather than in patients with age >50 years (WMD = −1.06 mg/dL, 95%CI: [−6.92, 4.80], *p* = 0.723; I^2^ = 76.1%, *p* = 0.015) (Figure 4). Further subgroup analyses based on BMI, intervention duration, and control group type, as well as the results of meta-regression analyses, are presented in the Subgroup analysis and meta-regression analysis section and Table 2.

**FIGURE 3:**
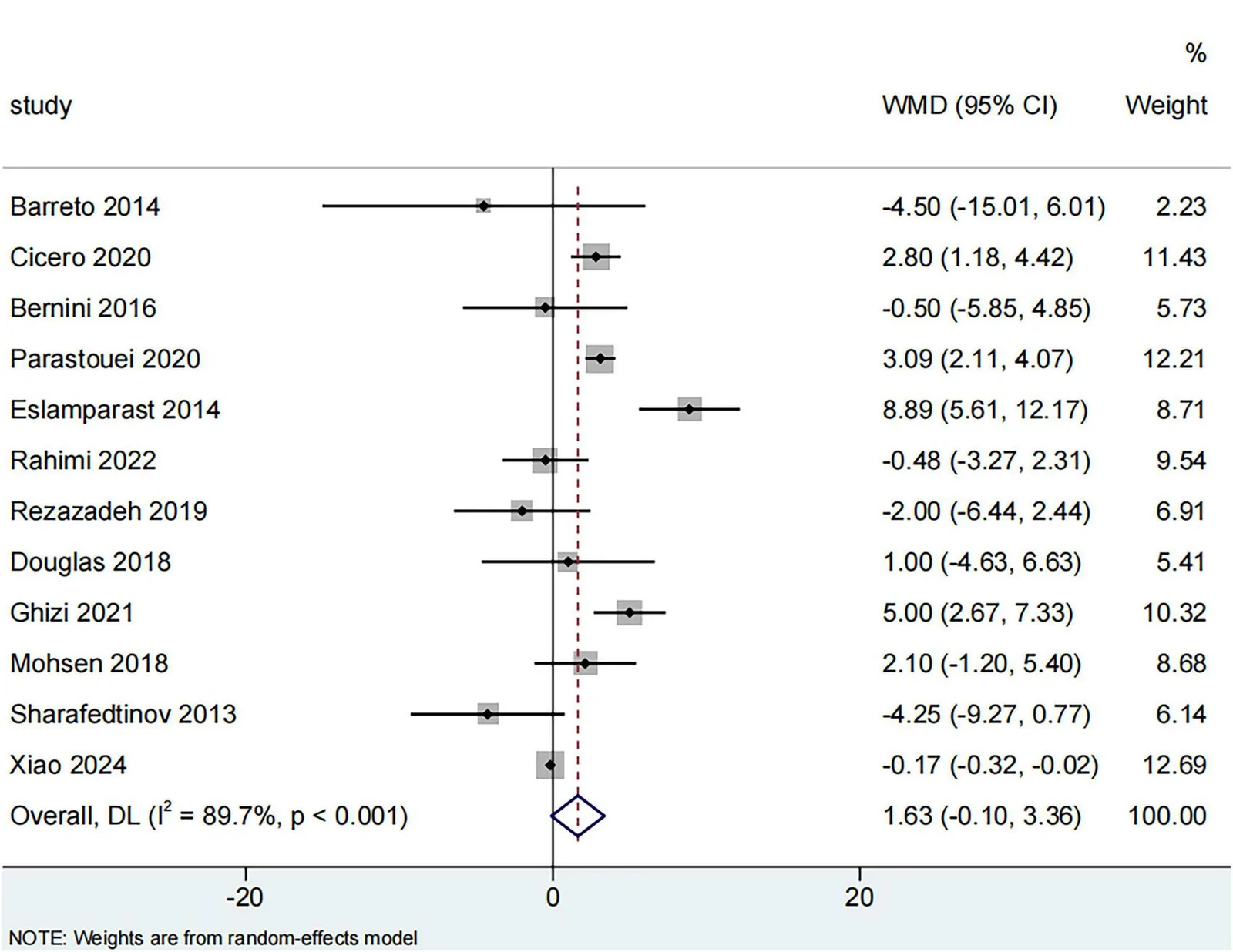
Forest plot of the HDL (overall).

**FIGURE 4:**
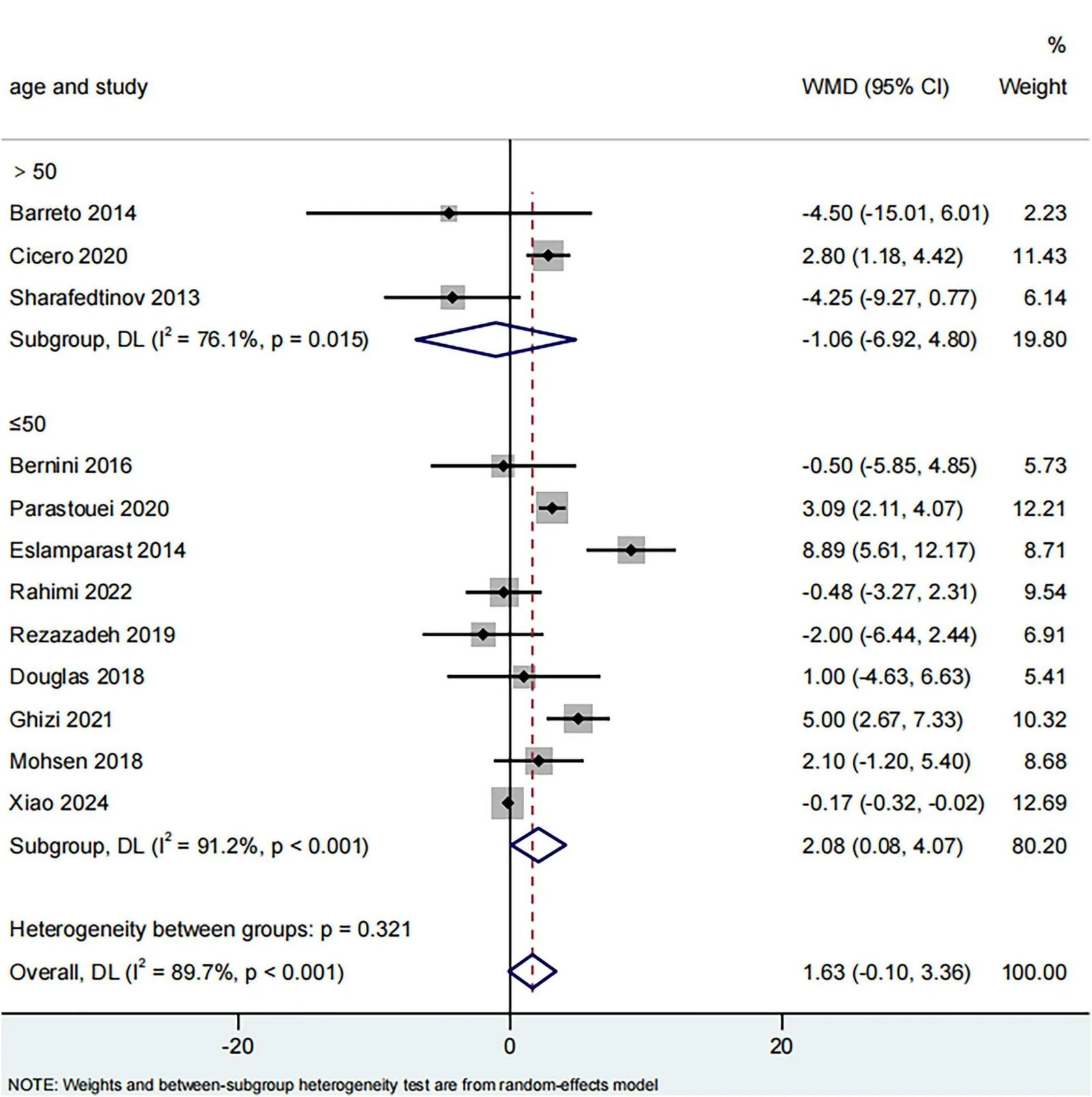
Forest plot of the HDL (Subgroup analysis based on age).

**TABLE 2:** Subgroup analysis and meta-regression analysis.

Outcome	Subgroup	Level	MD	LCI	UCI	p_sub	*z*	*p*
Glucose	Age	≤50	−0.541	−1.135	0.054	0.592	−0.536	0.592
		>50	−1.149	−3.293	0.995	0.592		
	BMI	≤30	−0.585	−1.176	0.006	0.992	0.01	0.992
		>30	−0.572	−2.899	1.754	0.992		
	CG	NI	3.500	−4.073	11.073	0.570	−0.801	0.423
		NP	−0.569	−4.801	3.664	0.570		
	Time	Placebo	−0.608	−1.188	−0.029	0.570	0.465	0.642
		≤2 months	−0.982	−2.753	0.789	0.642		
		>2 months	−0.538	−1.143	0.068	0.642		
HDL	Age	≤50	−0.052	−0.201	0.097	0.011	−1.038	0.299
		>50	1.915	0.400	3.431	0.011		
	BMI	≤30	−0.145	−0.296	0.006	0.000	0.478	0.633
		>30	2.827	2.062	3.591	0.000		
	CG	NI	1.000	−4.629	6.629	0.057	0.983	0.326
		NP	1.831	0.277	3.384	0.057		
	Time	Placebo	−0.051	−0.200	0.098	0.057	−0.267	0.789
		≤2 months	2.881	2.110	3.651	0.000		
		>2 months	−0.145	−0.297	0.006	0.000		
LDL	Age	≤50	−0.520	−1.098	0.058	0.023	−1.242	0.214
		>50	−6.954	−12.458	−1.451	0.023		
	BMI	≤30	−0.462	−1.051	0.128	0.051	−0.809	0.419
		>30	−3.139	−5.763	−0.516	0.051		
	CG	NI	1.500	−18.684	21.684	0.199	0.462	0.644
		NP	−6.821	−13.687	0.045	0.199		
	Time	Placebo	−0.548	−1.125	0.029	0.199	0.401	0.688
		≤2 months	−2.920	−5.548	−0.293	0.075		
		>2 months	−0.473	−1.062	0.116	0.075		
Total cholesterol	Age	≤50	0.117	−0.490	0.725	0.134	−0.611	0.541
		>50	−5.778	−13.463	1.907	0.134		
	BMI	≤30	0.117	−0.499	0.734	0.534	0.425	0.671
		>30	−0.926	−4.160	2.307	0.534		
	CG	NI	29.000	3.176	54.824	0.000	−1.624	0.104
		NP	21.996	14.252	29.740	0.000		
	Time	Placebo	−0.070	−0.678	0.537	0.000	−0.56	0.575
		≤2 months	−1.320	−4.515	1.876	0.382		
		>2 months	0.133	−0.484	0.749	0.382		
Triacyl	Age	≤50	0.030	−0.613	0.674	0.128	1.215	0.225
		>50	−7.725	−17.681	2.230	0.128		
	BMI	≤30	0.139	−0.507	0.785	0.000	−0.633	0.526
		>30	−13.184	−19.434	−6.935	0.000		
	CG	NI	18.574	−16.324	53.473	0.024	−0.576	0.564
		NP	−19.772	−35.130	−4.414	0.024		
	Time	Placebo	0.026	−0.617	0.670	0.024	−0.741	0.459
		≤2 months	−10.968	−16.662	−5.274	0.000		
		>2 months	0.140	−0.507	0.786	0.000		

NI, No intervention control; NP, Non-probiotic dairy control

#### LDL

Ten RCTs and one retrospective trial reported the effect of probiotic supplementation on the LDL level. The results indicate that the LDL level was significantly lower with probiotic supplementation than that in the placebo group (WMD = −0.59 mg/dL, 95%CI: [−1.17, −0.02], *p* = 0.044; I^2^ = 33.4%, *p* = 0.123) (Figure 5), having low heterogeneity.

**FIGURE 5:**
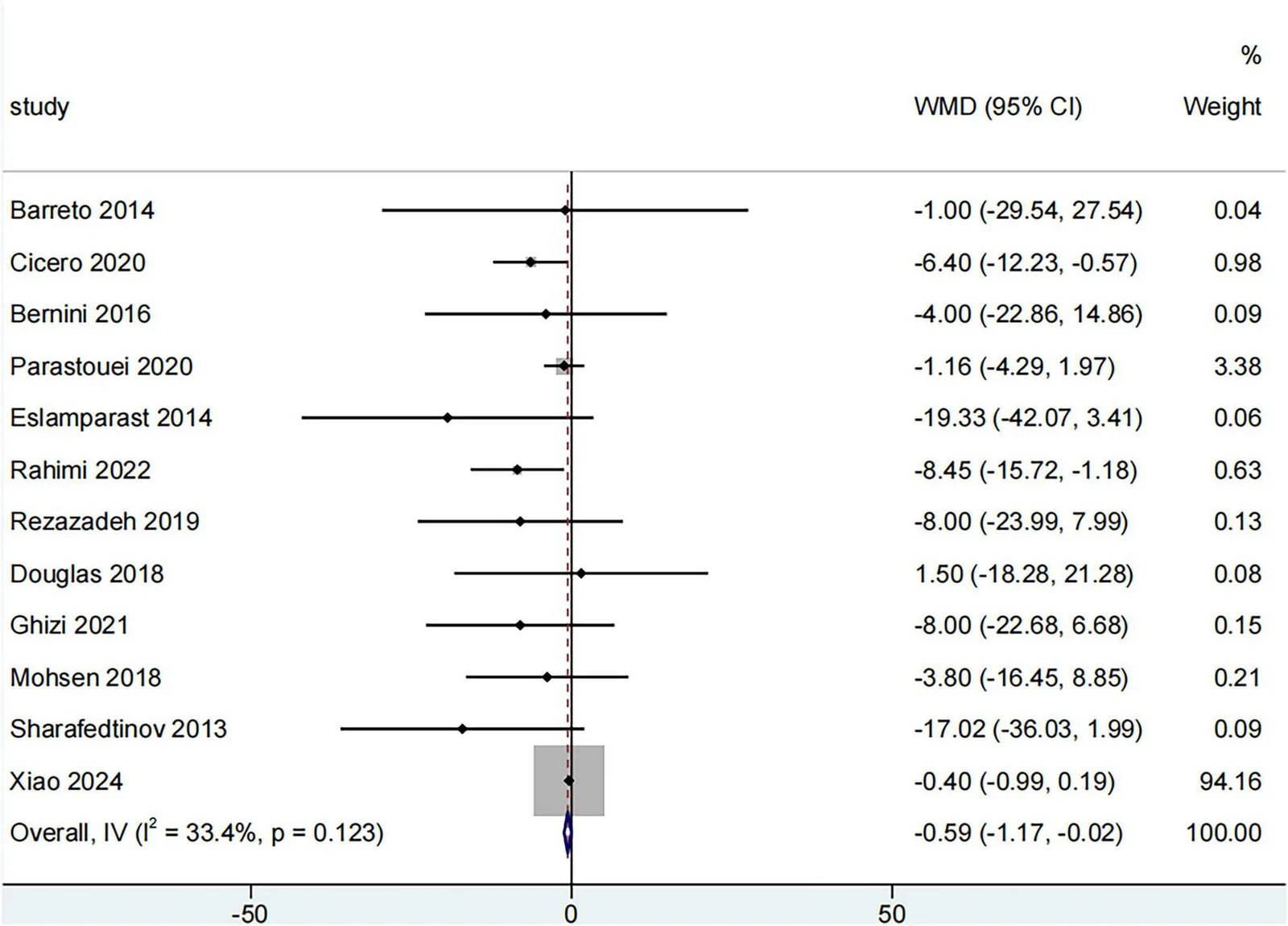
Forest plot of the LDL (overall).

#### Total cholesterol

Ten RCTs and one trial reported the effect of probiotic supplementation on the total cholesterol. The results indicate that there was no significant difference in total cholesterol between two groups (WMD = 1.80 mg/dL, 95%CI:[−5.37, 8.98], *p* = 0.622; I^2^ = 87.4%, *p* < 0.001) (Figure 6), having high heterogeneity. Subsequently, a subgroup analysis of the effect of probiotic supplementation on the total cholesterol was performed according to age. Three studies reported the age >50 years, and eight studies reported age ≤50 years. For age >50 years, probiotic supplementation reduced the total cholesterol (WMD = −5.84 mg/dL, 95%CI: [−13,47, 1.79], *p* = 0.134; I^2^ = 0.00%, *p* = 0.771), but having no significance. For age ≤50 years, probiotic supplementation exerted no significantly effect on the total cholesterol (WMD = 4.25 mg/dL, 95%CI: [−4.41, 12.90], *p* = 0.336; I^2^ = 90.5%, *p* < 0.001) (Figure 7).

**FIGURE 6:**
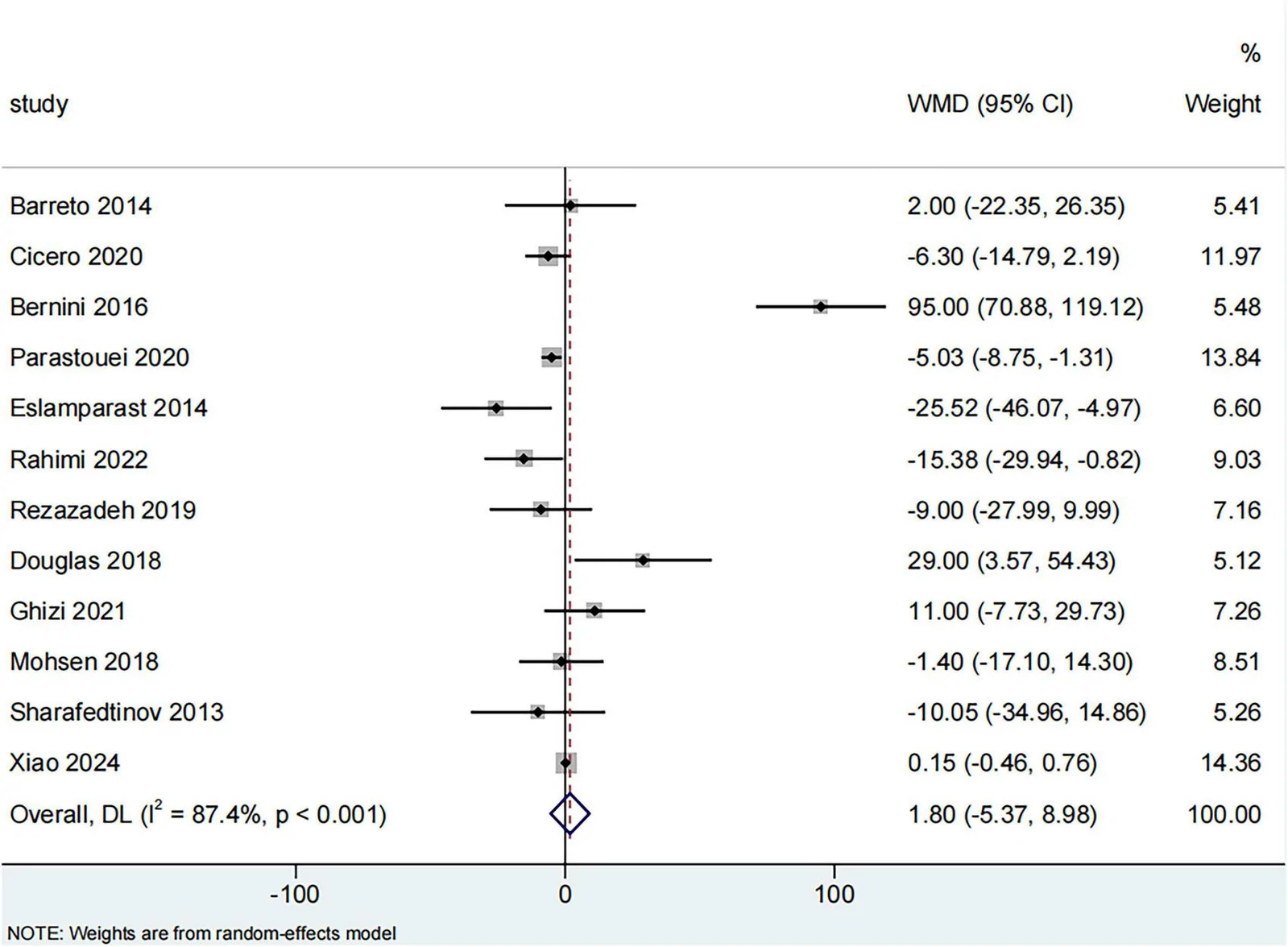
Forest plot of the total cholesterol (overall).

**FIGURE 7:**
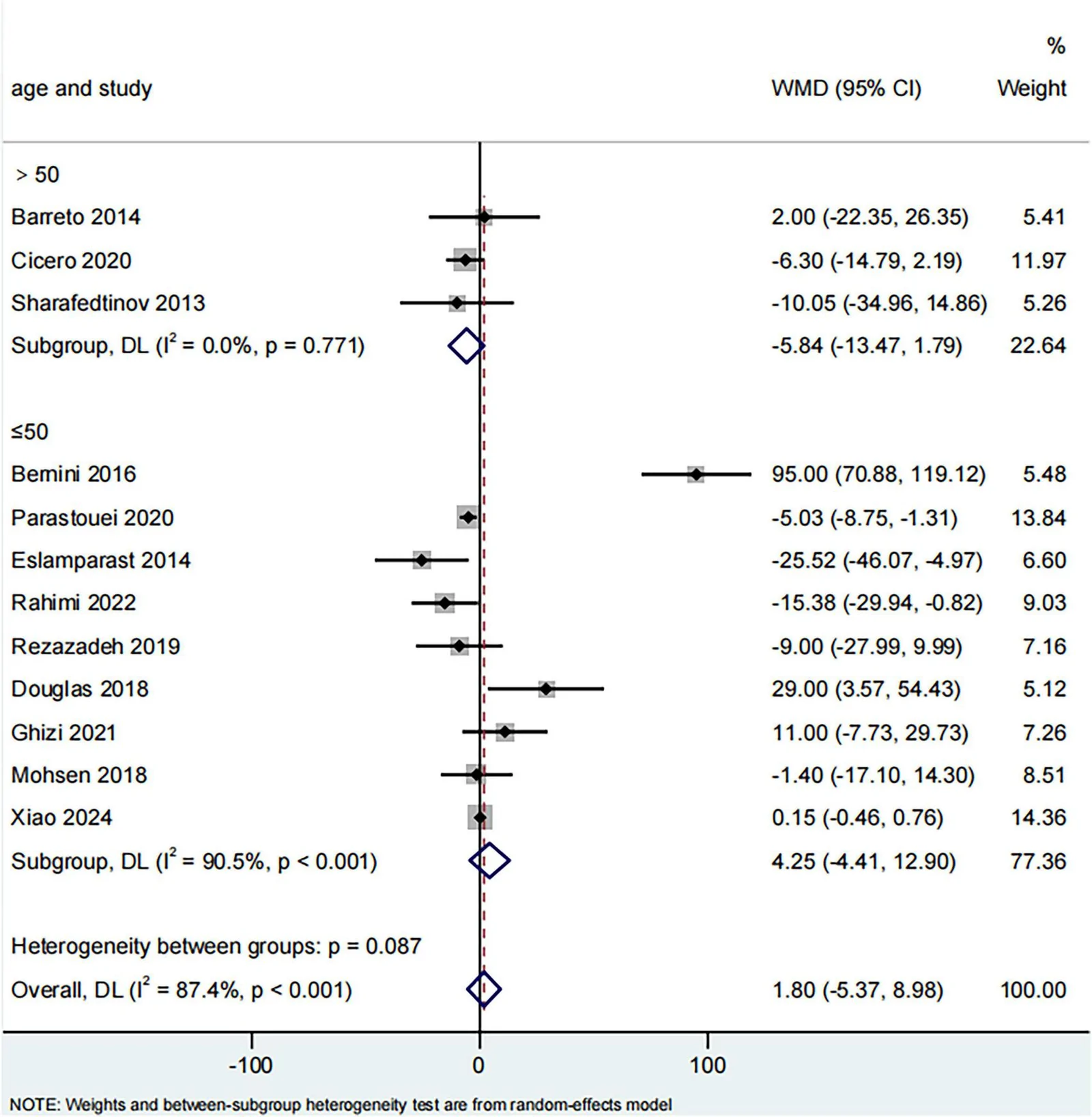
Forest plot of the total cholesterol (Subgroup analysis based on age).

### Triacylglycerol

Ten RCTs and one trial reported the effect of probiotic supplementation on the triacylglycerol. The results indicate that there was no significant difference in triacylglycerol between two groups (WMD = −7.58 mg/dL, 95%CI: [−17.15, 1.99], *p* = 0.121; I^2^ = 73.2%, *p* < 0.001) (Supplementary Figure 5), having high heterogeneity. Subsequently, a subgroup analysis of the effect of probiotic supplementation on the triacylglycerol was performed according to age. Four studies reported the age >50 years, and seven studies reported age ≤50 years. For age >50 years, probiotic supplementation exerted no significantly effect on triacylglycerol (WMD = 18.15 mg/dL, 95%CI: [−19.06, 55.36], *p* = 0.339; I^2^ = 59.8%, *p* = 0.059). However, for age ≤50 years, probiotic supplementation reduced serum triacylglycerol levels(WMD = −10.75 mg/dL, 95%CI:[−22.17, 0.67], *p* = 0.065; I^2^ = 77.8%, *p* < 0.001) (Supplementary Figure 6).

### Publication bias

The study used the funnel plot and Begg’s test to evaluate the publication bias in this meta-analysis (Supplementary Figures 7–11). No publication bias existed in glucose (Begg’s test, *p* = 0.756), HDL (Begg’s test, *p* = 0.304), LDL (Begg’s test, *p* = 1.000), total cholesterol (Begg’s test, *p* = 0.451) and triacylglycerol (Begg’s test, *p* = 0.213).

### Sensitivity analysis

The sensitivity analyses suggested that the glucose, HDL, LDL, total cholesterol and triacylglycerol were robust (Supplementary Figures 12–16).

### Subgroup analysis and meta-regression analysis

To further explore the potential sources of heterogeneity, subgroup analyses and meta-regression analyses were performed based on prespecified study characteristics, including age, BMI, intervention duration, and control group type. The effects of these variables on the pooled estimates were assessed to determine whether they contributed to the observed heterogeneity. Overall, none of these factors significantly influenced the pooled effect estimates for Glucose, HDL, LDL, or Triacyl (all P for subgroup differences > 0.05). Similarly, no significant associations were observed between these moderators and treatment effects in meta-regression analyses. For Total cholesterol, a significant difference was observed among different control group types (*P* < 0.001), with the largest effect estimate observed in the non-intervention group, followed by the non-probiotic control group, whereas the placebo-controlled studies showed a negative effect estimate. Other subgroup factors, including age, BMI, and intervention duration, did not significantly explain the observed heterogeneity (Table 2).

## Discussion

This meta-analysis of 12 high-quality CTs (633 patients) confirms that probiotic supplementation significantly improves lipid metabolism in Met patients (increased HDL-C, decreased LDL-C) and has distinct age specificity – beneficial effects on TG and HDL-C are limited to patients ≤50 years, with no significant effects in those >50 years. Probiotics show a slight hypoglycemic trend but no significant effect on TC, with high heterogeneity in TC outcomes. No publication bias was found, and results were robust. It should also be noted that obesity- and blood pressure-related outcomes were not included in the quantitative synthesis because these variables were inconsistently reported across the eligible studies, precluding reliable pooled analyses. Previous systematic reviews evaluating these outcomes have suggested that probiotic supplementation may exert modest beneficial effects on body weight, waist circumference, and blood pressure; however, these findings were beyond the scope of the present study.

### Probiotics may improve lipid metabolism in met patients: mechanistic insights and clinical implications

The effect of probiotics on improving HDL-C and reducing LDL-C levels in MetS patients is consistent with the results of some existing studies (15, 23), and its underlying mechanism is closely related to the regulation of gut microbiome and lipid metabolism by probiotics. On the one hand, probiotics (e.g., Lactobacillus and Bifidobacterium) may compete with intestinal pathogenic bacteria for nutrients and ecological niches, regulate the composition of gut microbiome, increase the abundance of SCFA-producing bacteria, and SCFA can inhibit hepatic cholesterol synthesis and promote reverse cholesterol transport (RCT) (24), thus increasing HDL-C levels-the “good cholesterol” that is responsible for RCT, anti-inflammation and endothelial protection (25). On the other hand, probiotics may produce bile salt hydrolase (BSH), which decomposes intestinal bile salts, reduces the reabsorption of bile acids, and promotes the liver to use cholesterol to synthesize new bile acids, thus reducing the level of LDL-C in the blood (24). In addition, probiotics may inhibit the oxidation of LDL-C (26), and oxidized LDL-C (Ox-LDL) is an important biomarker of cardiovascular disease in MetS patients (27, 28), so the reduction of LDL-by probiotics may also reduce the risk of cardiovascular complications in Met patients. Notably, the LDL-lowering effect of probiotics in this study has low heterogeneity, indicating that this effect is stable and not affected by probiotic strains, intervention forms and study populations. This finding has important clinical implications: LDL-C is a core therapeutic target for Met and cardiovascular disease, and probiotic supplementation can be used as an adjuvant intervention measure for Met patients with elevated LDL-C, especially for patients who cannot tolerate lipid-lowering drugs or have mild dyslipidemia.

The lack of significant improvement in fasting glucose may be explained by several factors. Glucose homeostasis is regulated by multiple physiological pathways, and the relatively short intervention duration, together with the mildly elevated baseline glucose levels in most MetS patients, may have limited the detectable effect of probiotic supplementation (29). Likewise, no significant reduction in total cholesterol was observed, which may be attributed to the heterogeneity of probiotic strains, intervention duration, and dosage among the included studies. In addition, total cholesterol reflects the combined concentrations of different lipoprotein fractions; therefore, the increase in HDL-C and decrease in LDL-C may partly offset changes in total cholestero (30).

### Age specificity of probiotic efficacy

The age-specific effects are the key novel finding, explaining inconsistencies in previous research (negative studies focus on the elderly, positive on young/middle-aged). However, these findings should be interpreted cautiously because the age cutoff was exploratory and the number of studies within each subgroup was limited. Three main reasons underpin this: (1) Gut microbiome aging: Gut microbiome diversity and probiotic strain abundance (Lactobacillus/Bifidobacterium) decrease with age, making exogenous probiotics hard to colonize in the elderly; young/middle-aged patients have a more flexible microbiome that readily adapts to probiotics. (2) Metabolic characteristic differences: Young/middle-aged Met patients have mild, reversible metabolic disorders linked to unhealthy lifestyles (no severe organ damage/comorbidities), while the elderly have long disease courses, multiple comorbidities, and age-related metabolic decline, blunting probiotic effects. (3) Intervention scheme mismatch: Fixed probiotic doses/durations ignore age-related differences in intestinal absorption; elderly patients have reduced intestinal peristalsis and digestive juice secretion, lowering probiotic survival and effective dose. To further investigate potential sources of heterogeneity, subgroup analyses and meta-regression analyses were conducted based on age, BMI, intervention duration, and control group type. Overall, these factors did not significantly modify the effects of probiotics on most metabolic outcomes. Neither age, BMI, intervention duration, nor control group type was significantly associated with changes in glucose, HDL-C, LDL-C, or triacylglycerol levels in meta-regression analyses. However, a significant difference was observed among different control group types for total cholesterol (*P* < 0.001), suggesting that comparator selection may partly contribute to the heterogeneity observed in TC outcomes. Nevertheless, these findings should be interpreted cautiously because subgroup analyses may have limited statistical power, particularly when the number of studies within individual subgroups is small. The high heterogeneity of probiotic intervention schemes is the main limitation of existing studies and the core reason for the inconsistency of research results. Based on the results of this study and the current research status, this study puts forward the following targeted solutions for the high heterogeneity of probiotic intervention schemes, which can be used for the design of subsequent clinical trials and meta-analyses: (1) Stratified research: Analyze single strains (e.g., Lactobacillus plantarum) and standardized synbiotics separately to clarify strain-specific efficacy, (2) Unify doses/concentrations: Adopt a unified viable cell concentration (e.g., 10^9^–10^10^ CFU/d) and dose based on weight/BMI.

It is necessary to consider the limitations of the present meta-analysis while interpreting the results. First, although 12 studies were included, the total sample size is relatively small (633 patients), and some subgroup analyses have a small sample size, which may lead to insufficient statistical power. Second, the probiotic intervention schemes of the included studies are diverse, and even after subgroup analysis based on age, there is still a certain degree of heterogeneity, which may affect the interpretation of the results. Third, this study did not include blood pressure and waist circumference, the core diagnostic indicators of Mets, due to the lack of unified reporting of these indicators in the included studies, so the effect of probiotics on the overall diagnostic indicators of MetS cannot be comprehensively evaluated. Fourth, this study did not analyze the effect of different probiotic intervention durations and doses due to the limited data of the included studies, and the optimal intervention scheme of probiotics for Met remains unclear. Future research should focus on large-sample, multi-center, double-blind CTs with standardized probiotic strains, doses, and durations; explore age-specific optimal intervention schemes and critical age thresholds; combine multi-omics to clarify molecular mechanisms: include all core Met indicators for comprehensive evaluation; and investigate synergistic effects of probiotics with lifestyle modification and conventional drugs. This will provide more precise evidence-based medicine for probiotic use in Met treatment.

In summary, this meta-analysis suggests that probiotic supplementation may provide modest beneficial effects on selected metabolic parameters in patients with metabolic syndrome. Specifically, probiotics were associated with increased HDL-C levels and reduced LDL-C levels, while the effects on fasting glucose and total cholesterol were not statistically significant. Although subgroup analyses suggested that patients aged ≤50 years may experience greater improvements in HDL-C and triglyceride levels, these findings should be interpreted cautiously because subgroup analyses were exploratory and the number of available studies was limited. Therefore, probiotic supplementation may be considered as a potential adjunctive strategy for metabolic management, particularly in patients with dyslipidemia; however, further large-scale randomized controlled trials are required to confirm age-specific effects and determine the optimal target population.

## Data Availability

The original contributions presented in the study are included in the article/Supplementary material, further inquiries can be directed to the corresponding author.

## Supplementary material

Supplementary Figure 1 Quality assessment of RoB 2.0 Tool (High risk proportions).

Supplementary Figure 2 Quality assessment of RoB 2.0 Tool (Assessment matrix per study).

Supplementary Figure 3 Quality assessment of ROBINS-I (High risk proportions).

Supplementary Figure 4 Quality assessment of ROBINS-I (Assessment matrix per study).

Supplementary Figure 5 Forest plot of the triacylglycerol (overall).

Supplementary Figure 6 Forest plot of the triacylglycerol (Subgroup analysis based on age).

Supplementary Figure 7 Funnel plot of glucose.

Supplementary Figure 8 Funnel plot of HDL.

Supplementary Figure 9 Funnel plot of LDL.

Supplementary Figure 10 Funnel plot of total cholesterol.

Supplementary Figure 11 Funnel plot of triacylglycerol.

Supplementary Figure 12 Leave-one-out analysis of glucose.

Supplementary Figure 13 Leave-one-out analysis of HDL.

Supplementary Figure 14 Leave-one-out analysis of LDL.

Supplementary Figure 15 Leave-one-out analysis of total cholesterol.

Supplementary Figure 16 Leave-one-out analysis of triacylglycerol.
