# Supplementary material for: The clinical effects of probiotic supplementations on metabolic syndrome: a systematic review and meta-analysis
Source: Front Nutr. 2026 Sep 14;13:1888320. doi: 10.3389/fnut.2026.1888320 (PMC13616635; doi:10.3389/fnut.2026.1888320)
Supplement: Supplementary file 1 [file Supplementary_file_1.docx]

Supplementary Figure S15. Leave-one-out analysis of total cholesterol.

Supplementary Figure S16. Leave-one-out analysis of triacylglycerol.
