# Supplementary material for: The clinical effects of probiotic supplementations on metabolic syndrome: a systematic review and meta-analysis
Source: Front Nutr. 2026 Sep 14;13:1888320. doi: 10.3389/fnut.2026.1888320 (PMC13616635; doi:10.3389/fnut.2026.1888320)
Supplement: Supplementary file 2 [file Table_1.docx]

**Supplementary Table 1 Search Strategy**

**Search strategy of PubMed**

| NO. | Search Details | Results |
| --- | --- | --- |
| #6 | (#1 OR #2) AND (#3 OR #4) Filter: Humans | 1,131 |
| #5 | (#1 OR #2) AND (#3 OR #4) | 2,027 |
| #4 | (((((((((((((((Metabolic Syndromes) OR (Metabolic Syndrome X)) OR (Insulin Resistance Syndrome X)) OR (Metabolic X Syndrome)) OR (Dysmetabolic Syndrome X)) OR (Reaven Syndrome X)) OR (Metabolic Cardiovascular Syndrome)) OR (Cardiometabolic Syndrome)) OR (Cardiometabolic Syndromes)) OR (insulin resistance syndrome)) OR (metabolic syndrome)) OR (MetSyn)) OR (Reaven syndrome)) OR (Reaven`s syndrome)) OR (Reaven`s syndrome X)) OR (syndrome of insulin resistance) | 256,413 |
| #3 | "Metabolic Syndrome"[Mesh] | 43,769 |
| #2 | ((Probiotic) OR (probiotics)) OR (probiotic agent) | 65,535 |
| #1 | "Probiotics"[Mesh] | 33,933 |

**Search strategy of EMBASE**

| No. | Query | Results |
| --- | --- | --- |
| #6 | #5 AND 'human'/de | 1,281 |
| #5 | (#1 OR #2) AND (#3 OR #4) | 1,535 |
| #4 | 'metabolic syndromes':ti,ab,kw OR 'metabolic syndrome x':ti,ab,kw OR 'insulin resistance syndrome x':ti,ab,kw OR 'metabolic x syndrome':ti,ab,kw OR 'dysmetabolic syndrome x':ti,ab,kw OR 'reaven syndrome x':ti,ab,kw OR 'metabolic cardiovascular syndrome':ti,ab,kw OR 'cardiometabolic syndrome':ti,ab,kw OR 'cardiometabolic syndromes':ti,ab,kw OR 'insulin resistance syndrome':ti,ab,kw OR 'metabolic syndrome':ti,ab,kw OR 'metsyn':ti,ab,kw OR 'reaven syndrome':ti,ab,kw OR 'reaven`s syndrome':ti,ab,kw OR 'reaven`s syndrome x':ti,ab,kw OR 'syndrome of insulin resistance':ti,ab,kw | 129,573 |
| #3 | 'metabolic syndrome'/exp | 125,582 |
| #2 | 'probiotic':ti,ab,kw OR 'probiotics':ti,ab,kw OR 'probiotic agent':ti,ab,kw | 72,671 |
| #1 | 'probiotic agent'/exp | 77,826 |

**Search strategy of Cochrane Library**

| NO. | Search deatiles | Hits |
| --- | --- | --- |
| #1 | MeSH descriptor: [Probiotics] explode all trees | 4,066 |
| #2 | (Probiotic):ti,ab,kw OR (probiotics):ti,ab,kw OR (probiotic agent):ti,ab,kw | 12,750 |
| #3 | MeSH descriptor: [Metabolic Syndrome] explode all trees | 3,828 |
| #4 | (Metabolic Syndromes):ti,ab,kw OR (Metabolic Syndrome X):ti,ab,kw OR (Insulin Resistance Syndrome X):ti,ab,kw OR (Metabolic X Syndrome):ti,ab,kw OR (Dysmetabolic Syndrome X):ti,ab,kw OR (Reaven Syndrome X):ti,ab,kw OR (Metabolic Cardiovascular Syndrome):ti,ab,kw OR (Cardiometabolic Syndrome):ti,ab,kw OR (Cardiometabolic Syndromes):ti,ab,kw OR (insulin resistance syndrome):ti,ab,kw OR (metabolic syndrome):ti,ab,kw OR (MetSyn):ti,ab,kw OR (Reaven syndrome):ti,ab,kw OR (Reaven`s syndrome):ti,ab,kw OR (Reaven`s syndrome X):ti,ab,kw OR (syndrome of insulin resistance):ti,ab,kw | 15,123 |
| #5 | (#1 OR #2) AND (#3 OR #4) | 297 |

**Search strategy of Web of science**

| NO. | Search deatiles | Hits |
| --- | --- | --- |
| #1 | (TS=(Probiotic) OR TS=(probiotics)) OR TS=(probiotic agent) | 84,172 |
| #2 | ((((((((((((((TS=(Metabolic Syndromes) OR TS=(Metabolic Syndrome X)) OR TS=(Insulin Resistance Syndrome X)) OR TS=(Metabolic X Syndrome)) OR TS=(Dysmetabolic Syndrome X)) OR TS=(Reaven Syndrome X)) OR TS=(Metabolic Cardiovascular Syndrome)) OR TS=(Cardiometabolic Syndrome)) OR TS=(Cardiometabolic Syndromes)) OR TS=(insulin resistance syndrome)) OR TS=(metabolic syndrome)) OR TS=(MetSyn)) OR TS=(Reaven syndrome)) OR TS=(Reaven`s syndrome)) OR TS=(Reaven`s syndrome X)) OR TS=(syndrome of insulin resistance) | 182,398 |
| #3 | #2 AND #1 | 1,599 |
